# An update of the consensus statement on insulin resistance in children 2010

**DOI:** 10.3389/fendo.2022.1061524

**Published:** 2022-11-16

**Authors:** Veronica Maria Tagi, Sona Samvelyan, Francesco Chiarelli

**Affiliations:** ^1^ Department of Pediatrics, University of Chieti, Chieti, Italy; ^2^ Paediatric Outpatient Department, Moscow, Russia

**Keywords:** insulin resistance, children, obesity, metabolic syndrome, genetics, diagnosis, treatment

## Abstract

In our modern society, where highly palatable and calorie-rich foods are readily available, and sedentary lifestyle is common among children and adolescents, we face the pandemic of obesity, nonalcoholic fatty liver disease, hypertension, atherosclerosis, and T2D. Insulin resistance (IR) is known to be the main underlying mechanism of all these associated health consequences; therefore, the early detection of IR is fundamental for preventing them.A Consensus Statement, internationally supported by all the major scientific societies in pediatric endocrinology, was published in 2010, providing all the most recent reliable evidence to identify the definition of IR in children, its measurement, its risk factors, and the effective strategies to prevent and treat it. However, the 2010 Consensus concluded that further research was necessary to assess some of the discussed points, in particular the best way to measure insulin sensitivity, standardization of insulin measurements, identification of strong surrogate biomarkers of IR, and the effective role of lifestyle intervention and medications in the prevention and treatment of IR.The aim of this review is to update each point of the consensus with the most recent available studies, with the goal of giving a picture of the current state of the scientific literature regarding IR in children, with a particular regard for issues that are not yet fully clarified.

## Insulin resistance refers to reduced whole body glucose uptake

Intake of carbohydrates and, therefore, elevated blood glucose levels lead to an activation of complex regulatory pathways, which are responsible for the clearance of glucose from the bloodstream and homeostatic control of blood glucose. The increased secretion of insulin by pancreatic beta cells is a direct response leading to enhanced glucose transport into peripheral tissues (1–3). Insulin resistance (IR) is the inverse state of insulin sensitivity (IS) (4) and the relative unresponsiveness of peripheral tissues to the effects of the hormone (2, 5). It can be described as impaired signal transduction and biological actions in response to increasing amounts of insulin in the circulation (6).

Peripheral IR contributes to the impairment of glucose disposition and IGT (7). IR is associated with reduced insulin-stimulated glucose utilization (8), primarily by the skeletal muscle and adipose tissue (1, 2, 4). In patients with type 2 diabetes (T2D), muscle IR is responsible for 85%–90% weakened total body glucose disposal (7).

IR is developed during some physiological conditions, such as pregnancy or puberty, as well as during intercurrent infections (8).

## Insulin resistance is a continuum

IR is a continuum from the stage of impaired IS to impaired glucose tolerance, followed by T2D (4, 7, 9). It is the earliest metabolic abnormality of T2D (7).

## Insulin resistance is commonly associated with obesity

The prevalence of IR is higher in overweight and obese children compared with normal-weight children (10).

Obesity is characterized by an excessive accumulation of adipose tissue in the body (3, 11). IR is the main factor for many of the complications of obesity (11). According to the World Health Organization (WHO), the number of obese children and adolescents has increased tenfold over the past four decades (12). 

## One of the consequences of insulin resistance is chronic compensatory hyperinsulinemia

In patients with IR, greater concentrations of insulin are needed to achieve the same effects that were earlier elicited by lower hormone levels (13). Primarily, pancreatic beta cells try to compensate metabolic dysregulated processes by increasing the secretion of insulin (8, 9, 14), but chronic hyperinsulinemia may play the crucial role in the pathology and progression of metabolic disorders (15). Hyperinsulinemia is accompanied by increased appetite and body weight gain (9). Ultimately, the pancreatic beta cell function declines because of glucolipotoxicity and genetic factors (14), and the secretion of insulin becomes insufficient (8, 9).

## Standards for insulin resistance in children, with definitions for normal and abnormal levels, are nonexistent

There is no standard definition of IS/IR in children (4, 13, 15, 16). The assessment of IS requires accurate measurement of insulin (17). However, there is not any standardized technique for the measurement of plasma insulin, and that makes it difficult to compare results between different laboratories (13).

Van der Aa et al. conducted a systematic review of all population-based studies reporting on the incidence of IR in children. In 18 population-based studies, six different methods were used to determine IR: homeostasis model assessment insulin resistance (HOMA-IR), fasted plasma insulin (FPI), the quantitative insulin sensitivity check index (QUICKI), the fasted glucose/insulin ratio (FGIR), HOMA2, and the McAuley index. The cutoff values used to define IR also varied (10). There is also no agreement regarding the necessity of using the age-adjusted cutoff points when determining IR in children (15, 18).

## The euglycemic hyperinsulinemic clamp is the “gold standard” for measuring insulin sensitivity; the frequently sampled IV glucose tolerance test and steady-state plasma glucose methods are also valid measurements

The HE-clamp, proposed by Andres et al. in 1966 and developed by DeFronzo et al. in 1979 (19), is the gold standard for the measurement of IS. This method evaluates insulin-mediated glucose utilization under steady-state conditions (20). However, the clamp method is invasive, time-consuming, and expensive, and, therefore, it cannot be used routinely in clinical practice (21).

In 1979, Bergman et al. proposed the minimal model methodology as a simple alternative to the HE-clamp. During the frequently sampled intravenous glucose tolerance test (FSIGT1), the dynamics of glucose and insulin are analyzed *via* a computer (22). Further studies showed that the IS index derived by the minimal model reliably correlates with the results of the clamp method (23).

## The homeostasis model assessment and the quantitative insulin-sensitivity check index do not offer any advantages over fasting insulin in euglycemic children

As the clamp method is not feasible on a large scale, several surrogate tools have been developed to evaluate IS. The most widely used surrogate markers are fasting surrogate indices such as fasting plasma insulin (FPI), HOMA-IR, and QUICKI (24). Several studies showed high correlation between these markers in children (15, 25–27).

George et al. evaluated the correlations between the clamp-measured IS and the surrogate estimates in 188 obese and overweight adolescents with normal glucose tolerance, prediabetes, and diabetes. The results showed that fasting indices correlated significantly with clamp IS. OGTT-derived surrogates did not demonstrate any advantage over the fasting indices when compared with clamp methodology. Furthermore, HOMA or QUICKI did not show any advantage over the 1/I_F_ when compared with the clamp-method results (25).

Schwartz et al. compared the surrogate measures of IR (fasting insulin, HOMA, QUICKI, and FGIR) with the insulin clamp measure of IR in a large cohort of adolescents. The results showed that other surrogate measures seem not to have any advantage over fasting insulin (26).

## Fasting insulin is a poor measure of whole-body insulin sensitivity in an individual child

Despite the aforementioned, fasting insulin is a poor marker of IS, as it does not always correlate well in children with the gold-standard clamp-measured IS (26, 27). This index may reveal compensatory hyperinsulinemia but could not be used as a marker of IR in a large clinical practice (24).

## The two most important biological conditions associated with insulin resistance in childhood are ethnicity and puberty

Transient reduction in whole-body IS and a temporary increase in IR are observed during puberty (7–9, 13, 28). During puberty, IS decreases by 25%–50% and recovers when pubertal development ends (29). Impaired IS during puberty is one of the main factors increasing the frequency of glucose metabolism disorders (30). The changes in hormonal profile during puberty are well known to affect IS, but the exact mechanisms of this are not fully understood so far (28). Increased growth hormone and IGF-1 secretion, accompanied by increased lipid breakdown and increased free fatty acids (FFAs) in the circulation, as well as rising concentrations of the sex hormone, contribute in developing IR during puberty (7, 28).

Another biological condition strongly associated with IR in children is race/ethnicity. Several studies compared IS in different ethnic groups and found ethnicity being an important risk factor for IR in children. It has been demonstrated that Hispanic, African-American, Asian, and Pima Indian children are more insulin-resistant compared with white children (31–33).

## Based on current screening criteria and methodology, there is no justification for screening children for insulin resistance, including obese children

It is well known that IR in children and adolescents may predict cardiovascular risk in the future (34). A strong association between IR and several chronic diseases makes the screening program for an early identification of at-risk children highly important. At the same time, the reliable methods of the measurement of IR are not available tools in a large clinical practice. There is no agreement regarding a uniform definition and cutoff value of IR (10), as well as no recommended pharmacological treatment for isolated IR in children (4); that is why screening programs for IR in children do not exist even in obese patients (4, 35).

## Obesity, particularly increased abdominal visceral adiposity, and nonalcoholic fatty liver disease are associated with insulin resistance in children

Nonalcoholic fatty liver disease (NAFLD) is associated with IR in children (4) and is the hepatic manifestation of metabolic syndrome (MetS) (36). It is the most common pediatric liver disease (37). NAFLD is the fatty infiltration of the liver in the absence of other causes of liver damage, such as genetic/metabolic disorders, alcohol consumption, infections, medications, or malnutrition (38). The pathogenesis of NAFLD is multicomponent (2, 39, 40). Hepatic triglyceride accumulation leads to steatosis (2). Excessive hepatic FFA accumulation or ‘lipotoxicity’ causes the activation of intracellular signaling pathways. Further hepatocellular damage is mediated by multiple factors: altered gut flora, stellate cell activation, increased mitochondrial permeability, adipocytokines, apoptosis, etc. (39, 40). IR plays a crucial role in the pathogenesis of NAFLD by facilitating lipogenesis in hepatocytes, impairing the inhibition of lipolysis, and stimulating the secretion of adipokines and other cytokines (40).

## Polycystic ovary syndrome, independent of weight, is characterized by insulin resistance in childhood

PCOS is a heterogeneous disorder. The diagnostic criteria for adolescent girls of PCOS are menstrual irregularity, hyperandrogenemia, and/or clinical features of hyperandrogenism (41). PCOS is often associated with IR. The severity of IR is more pronounced in obese patients than in lean ones, both in adolescent girls (42, 43) and in women (44).

## Genetics and heritability play a role in childhood insulin resistance

Genome-wide association studies (GWASs) have identified several loci associated with IR (**Table 1** **)** (60).

**Table 1:** Variants associated with IR. Adapted from Brown and Yanovski (17).

SNP	Nearby gene	Chromosome	Ref
rs13081389	PPARG	3	(19, 45)
rs972283	KLF14	7	(46)
rs2943641	IRS1	2	(47)
rs780094	GCKR	2	(48)
rs8050136	FTO	16	(46, 49, 50)
rs7903146	TCF7L2	10	(51, 52)
rs1208	NAT2	8	(53)
rs6723108, rs998451	TMEM163	2	(54)
rs35767	IGF1	12	(48)
rs12970134	MC4R	18	(55, 56)
rs17046216	SC4MOL	4	(57)
rs7077836	TCERG1L	10	(57)
rs702634, rs4311394	ARL15	12	(58, 59)

The PPARγ (peroxisome proliferator-activated receptor gamma) variant Pro12Ala was first shown to be associated with a decreased risk of developing T2D (19, 45). rs972283 near KLF14 (kruppel-like factor 14) was identified through large-scale association analysis of a European cohort (46) associated with reduced insulin action (61). IRS1 (insulin receptor substrate 1) is a fundamental component of the insulin signaling pathway initiating the activation of PI3K in response to insulin (47). GCKR (glucokinase regulator) encodes glucokinase regulatory protein (GKRP), which inhibits the activity of glucokinase, an enzyme involved in the regulation of hepatic glucose disposal and storage (62). Studies on TCF7L2 (transcription factor 7-like 2) have not demonstrated its role in insulin-dependent tissues despite the most consistent association with the risk of developing T2D of any gene variants identified so far (51, 52, 63, 64). Moreover, a variant in NAT2 (N-acetyltransferase 2) was recently found to be a direct measure of IS (53).

Two loci near TMEM163 (transmembrane protein 163) were demonstrated to be associated with reduced plasma insulin and HOMA-IR (65). Low plasma levels of IGF-1 are known to be associated with a reduction in IS (54), and analysis of the rs35767 polymorphism indicates that carriers of the G allele have lower circulating levels of IGF-1 compared with A allele carriers (66). MC4R (melanocortin 4 receptor) was shown in a GWAS of a UK cohort of Indian-Asian and European ancestry to be associated with both IR and waist circumference, with higher risk-allele frequencies being found in the Indian-Asian cohort (67).

A GWAS of an African American cohort identified an SNP near TCERG1L (transcription elongation regulator 1-like) and one near SC4MOL (sterol-C4- methyl oxidase-like) as being associated with both fasting insulin and HOMA-IR; this finding was also present in a West African cohort (55). The exact function of ARL15 (ADP ribosylation factor like GTPase 15), involved in intracellular vesicle trafficking, is still unknown. Variants of these genes are associated with decreased adiponectin levels and associated with the risk of T2D, coronary heart disease, and higher HOMA-IR (57).

## Intrauterine exposure to poorly controlled maternal diabetes increases the risk of obesity, insulin resistance, and IGT in childhood

The offspring risk of T2D is more closely associated with maternal than paternal diabetes (68). The risk of T2D in the offspring is strongly associated with the diagnosis of diabetes in mothers before they reach the age of 50. This could be due both to genetic transmission of diabetes susceptibility and the intrauterine environment. In fact, women with GDM have been shown to have increased serum leukocyte count and levels of inflammatory cytokines, including interleukin (IL)-6 and tumor necrosis factor (TNF)-α, in comparison with control subjects. Exposure to a proinflammatory environment within the uterus may increase the fetus diabetes risk by influencing the fetal epigenome at this early stage; however, more studies are needed (69).

By now, there are no accepted treatments for GDM except for lifestyle intervention and insulin therapy in selected cases, whose effectiveness is limited due to frequent IR. Results regarding glyburide and metformin are promising; however, their long-term safety needs to be deepened (70).

## Postnatal and childhood weight gain increase the risk of insulin resistance in normal-birth-weight and small-for-gestational-age children

It is well known that postnatal weight gain is associated with IR risk and greater adiposity in children and young adults (4, 71–75) and predicts IR-related outcomes in adults (76, 77).

Ong et al. (78) showed that rapid weight gain from age 0 to 2 years, whether with or without prior fetal growth deceleration, was associated with increased HOMA-IR and systolic and diastolic blood pressure, manifesting as early as 3 years of age.

Adolescents are another category at risk of developing IR (7) because, during puberty, there is a surge in growth hormone and insulin-like growth factor-I (IGF-1), which causes IR increase through increased lipid breakdown and increased FFAs (79). Girls are more likely to develop T2D than boys (80): estrogen overstimulates insulin receptors on β-cells resulting in excessive insulin signaling and β-cell exhaustion (81). Obese children who develop T2D around puberty and continue to gain weight have a higher risk of persistent IR even post-puberty.

## Insulin resistance is a risk factor for prediabetes and T2D in childhood

According to Consensus 2020, IR and impaired-cell function are the two key components in the pathogenesis of T2D in youth (4). To support this statement, Kim et al. (82) conducted a cross-sectional study on 205 youth investigating the adipose IR index (adipose-IR) (calculated as fasting insulin × free fatty acids [FFAs]) across the spectrum from normal glucose tolerance (NGT) to IGT to T2D and the predictive power of adipose-IR for determining dysglycemia in youth. What emerged is that adipose-IR increases progressively along the spectrum of glucose tolerance from NGT to IGT to T2D and that adipose-IR is a significant predictor of dysglycemia.

## Insulin resistance is associated with the metabolic syndrome and cardiometabolic risk factors

IR is one of the main elements that define the MetS (83) and contribute to cardiovascular risk. Recently, some studies have directly demonstrated the association between IR and atherosclerotic abnormalities in children. Miniello et al. (84) showed a clear association between increased HOMA-IR and impaired endothelial function since childhood, correlating HOMA-IR with flow-mediated dilatation, carotid intima-media thickness, and the anteroposterior diameter of infrarenal abdominal aorta in children.

Moreover, the results from the Pune Children’s Study suggest that prepubertal glucose-insulin metabolism is associated with high markers of atherosclerosis in adulthood (85).

## Diet and weight loss drugs improve insulin sensitivity in adolescents through weight loss and other mechanisms

Although diet is well known to affect IS, it is unclear what dietary macronutrient composition could contribute more to the development of IR (86, 87).

High fiber intake contributes to reducing body weight and improving IS by slow gastric emptying and absorption of dietary carbohydrates and fats, increased satiety, and regulatory effect on inflammatory markers and gut hormones (88–90).

Fat intake is associated with lower IS, and this association in adolescents seems to be independent of body fat (91). Arslanian et al. demonstrated that increased fat/carbohydrate ratio is negatively correlated with IS and insulin clearance also in prepubertal children (32). In addition, in non-obese adolescents, consumption of a high-fat/low-carbohydrate diet, even during a short time (7 days), was shown to impair IS (92). Regarding the fat quality, a consistent differential effect has not been found (93).

IS could be impaired also by high-glycemic-index food intake (94); however, data regarding the efficacy of low-glycemic-index diet in reducing IR are controversial (95).

## Exercise and fitness improve insulin sensitivity through weight loss and also mechanisms independent of weight loss in adolescents

Exercise contributes to the improvement of IS through other mechanisms besides the effect of weight loss (88, 96–99). Randomized studies have clearly shown the beneficial effect of physical activity on fasting insulin levels in overweight children before weight loss was documented (100, 101).

Regarding the type of exercise to recommend, several studies have demonstrated significant beneficial effects of both aerobic and combined aerobic and resistance exercises in overweight children independently of changes in body weight and percent body fat (102–105). However, data regarding the efficiency of resistance training on IR in children and adolescents are debatable (106, 107).

## Multicomponent lifestyle intervention improves insulin sensitivity more than individual lifestyle components in adolescents

A multicomponent lifestyle approach could be more effective in improving IS than a simpler approach (88, 108, 109). Dietary intake, physical activity, and sleeping habits can affect IS, and each is individually linked to IR (110). Androutsos et al. (110) demonstrated that a lifestyle pattern consisting of high moderate-vigorous physical activity and frequent eating habit was inversely related to HOMA-IR. Patterns combining different lifestyle behaviors may be more practical for translating into recommendations and thus more useful for public health.

## Metformin improves insulin sensitivity in adolescence

Metformin reduces IR by decreasing fasting plasma glucose and insulin concentrations in adults. Moreover, it is an insulin sensitizer and likely acts in the gut lumen through multiple mechanisms. In non-diabetic obese adults, it reduces food intake, determining weight loss and the reduction of fasting plasma glucose, cholesterol, and insulin concentrations. Furthermore, metformin has been shown to improve the BMI, body fat composition, fasting glucose, insulin, glycated hemoglobin (HbA1c), IR expressed by the HOMA-IR, blood pressure, and lipid profile in short trials on small sample sizes of children and adolescents (111).

Metformin is approved by the US Food and Drug Administration (FDA) for the treatment of T2D in 10-year-old or older children, and it is the only treatment evaluated in children with prediabetes.

The 2017 Pediatric Obesity Clinical Guidelines by The Endocrine Society recommend using it only in selected patients, since its long-term benefits in insulin-resistant children are still to be analyzed (112).

## Maternal obesity, gestational diabetes, and smoking in pregnancy should be targeted to lessen obesity and insulin resistance in children

Infants of women with gestational diabetes (GDM) have an increased risk of becoming overweight or obese during adolescence and are more likely to develop type II diabetes later in life. Furthermore, according to several studies, epigenetic alterations of different genes of the fetus of a GDM mother *in utero* could result in the transgenerational transmission of GDM and type II diabetes (113).

A cross-sectional study conducted on 976 children and adolescents showed a significant association between maternal weight and cigarette consumption during pregnancy and the subsequent development of MetS in children (114).

Given these promises, the reinforcement of screening and prevention methods is a must.

## Breast-feeding should be promoted through public health interventions as a contributing factor to reducing the prevalence of obesity and potential insulin resistance later in life; in addition, ongoing dietary advice starting from weaning has the potential to prevent insulin resistance in the long term

Wisnieski et al. (115) conducted a systematic review on the effects of breastfeeding on the risk of MetS showing a protective role of breastfeeding on the development of MetS. Some studies also showed an inverse relationship between duration of breastfeeding and MetS (114, 116), whereas others did not find a temporal association (117, 118).

It has been postulated that, during pregnancy and the next 2 years of life, correct maternal eating habits, breastfeeding, and then weaning with an intake of correct doses of the components of diet are essential for reducing the risk of MetS (119).

## Identification of infants and preschool children at risk for obesity combined with intervention programs to prevent excessive weight gain should be developed and evaluated

Obesity is one of the leading causes of IR in childhood. Preventive strategies are the effective public health intervention in fighting against this pandemic. A multicomponent approach has been found beneficial in preventing obesity. All the environments where the child lives have a crucial role; therefore, family, school, and community should collaborate in the child’s healthy lifestyle. Furthermore, a structured weight reduction program individualized for every child is needed. Anti-obesity drugs are indicated only in rare specific cases, and bariatric surgery is recommended only in older adolescents, but data about its long-term effectiveness in this age group are limited (120).

## Conclusions

Since 2010, many further studies have been conducted to better explain the underlying mechanisms of IR and the characteristics of children with impaired IS. Although some concepts, such as the timing of rapid weight gain with respect to future IR and the role of IR in predicting the development of impaired glucose tolerance and T2D, have been better clarified, a validated definition of IR is still lacking. Further research is still needed to best measure IS and to find strong surrogate biomarkers of IR.

## Author contributions

VT and SS drafted the manuscript. FC provided critical feedback and helped in shaping the manuscript. All authors contributed to the article and approved the submitted version.

## Conflict of interest

The authors declare that the research was conducted in the absence of any commercial or financial relationships that could be construed as a potential conflict of interest.

## Publisher’s note

All claims expressed in this article are solely those of the authors and do not necessarily represent those of their affiliated organizations, or those of the publisher, the editors and the reviewers. Any product that may be evaluated in this article, or claim that may be made by its manufacturer, is not guaranteed or endorsed by the publisher.
